# Neurotoxicity and Mode of Action of *N, N*-Diethyl-*Meta*-Toluamide (DEET)

**DOI:** 10.1371/journal.pone.0103713

**Published:** 2014-08-07

**Authors:** Daniel R. Swale, Baonan Sun, Fan Tong, Jeffrey R. Bloomquist

**Affiliations:** University of Florida, Emerging Pathogens Institute, Department of Entomology and Nematology, Gainesville, Florida, United States of America; Rosalind Franklin University, United States of America

## Abstract

Recent studies suggest that *N, N*-diethyl-*meta*-toluamide (DEET) is an acetylcholinesterase inhibitor and that this action may result in neurotoxicity and pose a risk to humans from its use as an insect repellent. We investigated the mode of action of DEET neurotoxicity in order to define the specific neuronal targets related to its acute toxicity in insects and mammals. Although toxic to mosquitoes (LD_50_ ca. 1.5 µg/mg), DEET was a poor acetylcholinesterase inhibitor (<10% inhibition), even at a concentration of 10 mM. IC_50_ values for DEET against *Drosophila melanogaster*, *Musca domestica*, and human acetylcholinesterases were 6–12 mM. Neurophysiological recordings showed that DEET had excitatory effects on the housefly larval central nervous system (EC_50_: 120 µM), but was over 300-fold less potent than propoxur, a standard anticholinesterase insecticide. Phentolamine, an octopamine receptor antagonist, completely blocked the central neuroexcitation by DEET and octopamine, but was essentially ineffective against hyperexcitation by propoxur and 4-aminopyridine, a potassium channel blocker. DEET was found to illuminate the firefly light organ, a tissue utilizing octopamine as the principal neurotransmitter. Additionally, DEET was shown to increase internal free calcium via the octopamine receptors of *Sf*21 cells, an effect blocked by phentolamine. DEET also blocked Na^+^ and K^+^ channels in patch clamped rat cortical neurons, with IC_50_ values in the micromolar range. These findings suggest DEET is likely targeting octopaminergic synapses to induce neuroexcitation and toxicity in insects, while acetylcholinesterase in both insects and mammals has low (mM) sensitivity to DEET. The ion channel blocking action of DEET in neurons may contribute to the numbness experienced after inadvertent application to the lips or mouth of humans.

## Introduction

The insect repellent *N, N*-diethyl-*meta*-toluamide (DEET) is used more often than any other mosquito repellent, with over 200 million users, worldwide [1]. Because there is deliberate and widespread human exposure to DEET from its use as an insect repellent, questions have arisen regarding its toxicological profile and risk to humans. A recent study [2] suggested the toxic action of DEET may be due to an anticholinesterase effect, with implications for human safety. Large oral doses (blood concentration of 1 mmol/liter) of DEET lead to nausea, vomiting, bradycardia, and seizures [3], [4], as well as cardiotoxicity [5] in exposed humans. Contact exposure to DEET also has the potential for dermal effects, as it can lead to numbness and redness of the affected area [6]. The numbing sensation appears similar to that observed with local anesthetics, such as lidocaine, suggesting DEET might be acting on neuronal ion channels to yield an anesthetic-like effect.

DEET has insecticidal properties [7], [8], and was claimed to be synergized in its toxicity to German cockroaches by a compound (eserine) that also synergized the octopaminergic insecticides, chlordimeform (CDM) and amitraz [7]. However, data for DEET synergism by eserine were limited, and based solely upon the observation that 7 of 10 cockroaches were killed in a single combined dose test, making it impossible to assess statistical significance, since 95% confidence limits, slope, and probability values were absent. Moss [7] acknowledged that other mechanisms, such as augmented penetration, might contribute to DEET's synergistic interactions [7], while another study suggested that interaction with mixed function oxidases explained the synergism of DEET and the anticholinesterase insecticide, propoxur [9]. None of these studies [7]–[9] reported any direct mechanistic experiments.

**Figure 1:**
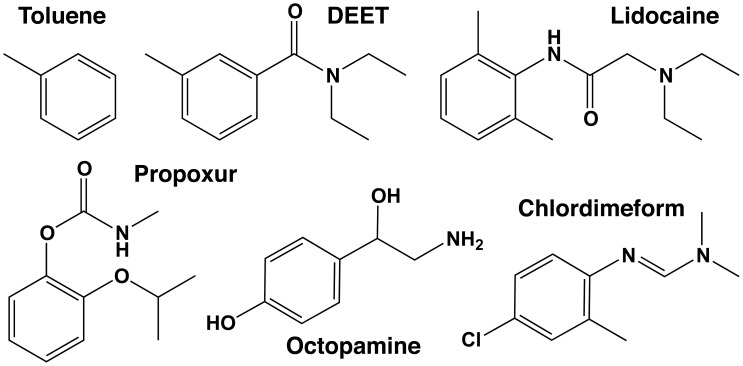
Chemical structures of the pharmacological agents used in this study.

The overall objectives of this study were to determine mechanisms underlying DEET neurotoxicity and compare its effects to known anticholinesterases (propoxur), local anesthetics (lidocaine and toluene), and octopaminergic compounds (octopamine and CDM), having some structural similarity to DEET (Fig. 1). In particular, we investigated the ability of DEET to mimic the neuroexcitatory action of octopamine [10], or the block of sodium or potassium channels by local anesthetics [11] and toluene [12], with the goal of providing insights into the specific targets of DEET underlying its acute toxicity to insects and mammals.

## Materials and Methods

### Compounds, Solvents, and Assay Reagents

DEET, lidocaine, toluene, cesium chloride, 4-aminopyridine (4-AP), and propoxur were all purchased from Sigma-Aldrich (St. Louis, MO, USA) and were ≥98% pure. The solvent dimethyl sulfoxide (DMSO) was used to dissolve agents when needed, and was also purchased from Sigma-Aldrich. Piperonyl butoxide (PBO, 90%) was obtained from Fairfield American Corporation (Frenchtown, NJ, USA) and CDM (97%) was from Nor-Am Chemical Company (Wilmington, DE, USA). Ellman assay [13] reagents are composed of sodium phosphate buffer, the enzyme substrate acetylthiocholine iodide (≥99% purity), and the reactive chromophore 5,5′-dithiobis-(2-nitro)benzoic acid (99% purity), all of which were purchased from Sigma-Aldrich.

### Enzymes, Insects, and Neuronal Cells

*In vitro* biochemical assays utilized five acetylcholinesterase (AChE) enzymes. Recombinant human AChE (*h*AChE) was purchased from a commercial source (lyophilized powder, Sigma C1682, St. Louis, MO, USA). Homogenates to prepare *Anopheles gambiae* AChE (*Ag*AChE) were obtained from the wild type G3 strain and the resistant AKRON strain (BEI Resources; http://www.beiresources.org/). AKRON mosquitoes (from Benin and donated by M. Akogbeto) have both knockdown resistance (*kdr*) to pyrethroids (L1014F sodium channel mutation) and the G119S AChE mutation (*ace*-1R), which confers resistance to carbamates [14], [15]. Both strains were cultured in the Emerging Pathogens Institute, University of Florida, Gainesville, FL, USA from eggs provided by the Center for Disease Control, Atlanta, GA, USA. Homogenates were also used to prepare *Aedes aegypti* AChE (*Ae*AChE) from mosquitoes provided by the CMAVE, USDA-ARS, Gainesville, FL, USA; *Musca domestica* AChE (*Md*AChE) from flies cultured in the Department of Entomology and Nematology, Medical Entomology Laboratory, University of Florida, Gainesville, FL, USA); and *Drosophila melanogaster* AChE (*Dm*AChE) from flies (Oregon-R strain) cultured in the Emerging Pathogens Institute, University of Florida, Gainesville, FL, USA. Homogenate enzymes were prepared from groups of ten non-blood fed adult female mosquitoes, five whole bodied *Drosophila*, or three *Musca* heads homogenized in 1 mL of ice-cold sodium phosphate buffer (0.1 M sodium phosphate, pH 7.8) with an electric motor driven glass tissue homogenizer. The homogenate was centrifuged at 5000×*g* using a Sorvall Fresco refrigerated centrifuge at 4 °C for 5 minutes. The supernatant was used as the enzyme source, and all enzyme preparations contained 0.3% (v/v) triton X-100 and 1 mg/ml BSA. Prior to use, the recombinant human enzyme was diluted 500-fold into sodium phosphate buffer containing triton X-100 and 1 mg/ml BSA.

Insects used for *in vivo* toxicity assays were obtained from the sources listed above. Rat neuronal cortex cells were purchased from Invitrogen (Grand Island, NY, USA), plated on 35 mm glass cover slips, and maintained in primary neuron basal medium without *L*-glutamine at 38 °C until used for patch clamp studies.

### Enzyme Inhibition Assays

Inhibition of AChE was determined using the Ellman assay [13] and was based on the method outlined in Carlier et al. [16]. Briefly, enzyme solution (10 µL) was added to each well of a 96-well micro assay plate, along with 20 µL of inhibitor in DMSO/saline suspension, and 150 µL of sodium phosphate buffer. Final concentration of DMSO was 0.1% in all incubations. The plate was incubated at 25 °C for ten minutes. Ellman assay reagents, acetylthiocholine iodide (0.4 mM, final concentration) and 5,5′-dithiobis-(2-nitro)benzoic acid (0.3 mM, final concentration), were prepared fresh and 20 µL was added to the enzyme to initiate the reaction. Changes in absorbance were recorded by a DYNEX Triad spectrophotometer (DYNEX Technologies, Chantilly, VA, USA) at 405 nm. Enzyme concentrations used were within the linear range, therefore eliminating the need for protein quantification. IC_50_ values (inhibitory concentration needed to inhibit 50% of the enzyme activity, negative Hill slope) were calculated using eight inhibitor concentrations by nonlinear regression to a four parameter logistic equation (variable Hill slope) using GraphPad Prism (GraphPad Software, San Diego, CA, USA).

### Toxicity Assays

Topical toxicity bioassays on adult female mosquitoes were performed based on the method of Pridgeon et al. [17]. Briefly, insects were chilled on ice for 3 min, during which the appropriate volume (200 nL for mosquitoes, 1 µL for *Musca*) of chemical (dissolved in 95% ethanol) was applied onto the abdomen using a handheld Hamilton microapplicator. For each inhibitor, five doses were applied to groups of ten insects each and repeated three times. An ethanol-only treatment was included in each experiment as a negative control. Insects were transferred into a holding chamber post-treatment and supplied with free access to sugar water for the duration of the experiment. Mortality was recorded at the 24-hour time point, pooled and analyzed by log-probit using Poloplus (LeOra Software Co., Petaluma, CA, USA) to determine 24 hr LD_50_ values (lethal dose needed to kill 50% of treated insects). Three LD_50_ values were obtained and the mean LD_50_ value was calculated and used for statistical analysis.

Diet toxicity assays were performed on mixed sex adult *Drosophila melanogaster* fasted for 8 hours prior to initiating the experiment. Flies were transferred into a container stoppered with a cotton ball infused with 1 ml of a sucrose/DEET solution containing 20 insects (2–3 day old adults). The assay was run three times on three different days with matched controls. Mortality was recorded at the 24-hour time point.

Contact toxicity bioassays were performed on both strains of *Anopheles gambiae*, G3 (susceptible) and AKRON (pyrethroid and anticholinesterase resistance) according to WHO protocol [18]. Adult female mosquitoes were 2–5 days of age and non-blood fed at the time of experimentation. Five concentrations of inhibitor dissolved in ethanol were prepared and treated by applying 2 mL of each concentration on a 180 cm^2^ (12 cm×15 cm) paper. Papers were left to dry for 24 hours prior to use. Mosquitoes were chilled for three minutes on ice, after which 25 females were placed in the WHO kit holding chamber to recover for one hour. Mosquitoes were then moved to the treatment chamber, which contain the treated paper, and exposed for one hour. After the one-hour exposure, the mosquitoes were transferred back to the holding chamber and maintained on 10% sugar solution for 24 hrs. Each concentration was repeated in triplicate. Mortality was recorded 24 hours post treatment and an LC_50_ (lethal concentration needed to kill 50% of exposed insects) was calculated using Poloplus (LeOra Software Company, Petaluma, CA, USA). Three LC_50_ values were obtained and the mean LC_50_ value was used for statistical analysis.

For all toxicity assays, control mortality was corrected for using Abbots formula [19]; where corrected percent mortality  =  (% alive in control–% alive in treated) /% alive in control.

### Electrophysiological Studies in Housefly

Neurophysiological recordings were performed on the central and peripheral nervous systems of larval *Musca domestica*. Glass pipette electrodes were pulled from borosilicate glass capillaries on a P-1000 Flaming/Brown micropipette puller (Sutter Instrument, Novato CA, USA). For CNS recordings [20], it was first excised from the larvae and placed in a separate dish with physiological saline (200 µL) containing: 157 mM NaCl, 3 mM KCl, 2 mM CaCl_2_, and 4 mM HEPES, pH = 7.25. The CNS was manually transected posterior to the cerebral lobes to disrupt the blood-brain barrier and enhance chemical penetration [20]. Any convenient peripheral nerve trunks were pulled into a recording suction electrode. Amplification and digitization of descending electrical activity originating from the CNS were converted on-line into a rate plot, expressed in Hz (MacLab analog to digital converter hardware and LabChart 7 software, ADInstruments, Colorado Springs, CO, USA). Activity was monitored for a ten minute time period to establish a constant baseline firing rate, as the spike frequency typically increased from 0 to 10 minutes before stabilization. After a baseline was established, the CNS preparation was directly exposed to test compound by adding 200 µL of solution to the bath containing 200 µL of saline. The final concentration of solvent in the bath was 0.1% DMSO. Each concentration was recorded for 3–5 min prior to the addition of the next drug concentration. Mean spike frequencies for each concentration were used to construct concentration-response curves to determine EC_50_ values (EC_50 = _ concentration of drug to elicit half maximal response, positive Hill slope), and were calculated by nonlinear regression (variable slope) using GraphPad Prism (GraphPad Software, San Diego, CA, USA) in a manner similar to that for AChE inhibition. Each drug concentration was replicated 3-5 times.

Neuromuscular recordings of the evoked excitatory postsynaptic potential (EPSP) were performed on third instar housefly larvae, essentially as described previously [21]. In short, the maggot was immobilized with pins, and the nervous and musculature systems were exposed. The preparation was then bathed in saline containing: 140 mM NaCl, 0.75 mM CaCl_2_, 5 mM KCl, 4 mM MgCl_2_, 5 mM NaHCO_3_, and 5 mM HEPES (pH = 7.25). The nerves were severed from the base of the CNS, which was removed, and a lateral nerve trunk innervating the longitudinal muscles was drawn into a suction electrode filled with saline. Stimuli were applied at 1 volt and of 0.2 sec duration to elicit a contraction from the longitudinal muscles. The stimulated muscle was then impaled with a recording glass capillary microelectrode filled with 1 M KCl to record effects on the evoked EPSP and membrane potential. Signals from all recordings were amplified and digitized with the use of the MacLab. Chemicals were applied to the preparation directly by hand pipetting 150 µL of solution into the bath volume of 150 µL.

### *In Vivo* Assay for Effects on Firefly Light Organ

Adult firefly beetles (*Photinus sp*.) were collected locally (Nashville, TN) and housed in well-ventilated chambers with tree leaves. The animals had free access to 10% sucrose solution. Chemicals were dissolved in absolute ethanol and 1 µL was topically applied directly to the ventral light organ with the use of a manual pipettor. Prior to DEET application, the beetles were immobilized with molten parafilm and were mounted on corrugated cardboard for the duration of the study. Due to the time required for bioactivation of CDM, insects were treated with 1 µL of CDM and were released back into the holding chamber for three hours. After three hours, they were mounted in the previously described manner and monitored for activation of the lantern. Photographs were captured with a handheld camera (Canon PowerShot SX110 IS). Pictures were captured within five minutes, four hours, and 10 minutes post application for DEET, CDM, and propoxur, respectively. DEET- and propoxur-treated insects were monitored for at least 1 hour post exposure to ensure the response was not slow acting. The original photographs were cropped and background shadows were enhanced to increase detail of the insect using Adobe Photoshop PS5 (Adobe Systems, San Jose, CA, USA). The color or brightness of the light organ was not manipulated in any way during post-processing of the image.

### Calcium Fluorescence Assay

The *Sf*21 insect cell line was obtained from a commercial supplier (Invitrogen, Carlsbad, CA, USA) and maintained in log phase culture in *Trichoplusia ni* Medium-Formulation Hink (TNM-FH) from Sigma-Aldrich (St. Louis, MO, USA), supplemented with 10% fetal bovine serum and 100 U/ml penicillin and streptomycin (Sigma-Aldrich). Cells were cultured in a non-humidified environment and maintained at 27°C. *Sf*21 cells were passaged every 3–5 days according to manufacturer's instructions, and maintained for about 30 passes before being replaced by fresh cells.

The FLIPR Calcium 5 Assay Evaluation Kit (Molecular Devices, Sunnyvale, CA, USA) was used to determine calcium fluorescence in *Sf*21 cells, a clonal line of *Sf*9 cells, which are known to possess octopamine receptors [22]. *Sf*21 cells were plated into 96-well black-wall, clear bottom plates 24 h before calcium fluorescence assay. Cell culture medium was carefully removed from each well of the plate, and 100 µL per well of Calcium 5 Assay buffer with 2.5 mM of probenecid and 1.3 mM calcium was then added. After 1 h incubation at room temperature, 100 µM of octopamine, DEET, or propoxur were applied to the assay plate, and fluorescence was measured immediately at 485 and 525 nm wavelengths for excitation and emission, respectively, with slits of 10 nm (excitation) and 20 nm (emission) on a SyntaxMax plate reader (BioTek, Winooski, VT, USA). The octopamine receptor antagonist phentolamine (100 µM) was co-incubated with cells for 1 h before drug challenge. Phentolamine, octopamine, and DEET were dissolved in water, while propoxur was dissolved in 100% DMSO to make a stock solution, and in the assay, DMSO was kept to 0.1%. The experiment was repeated in triplicate with different plates of cells to obtain means and SEMs for each experimental condition.

### Patch Clamp Recordings

Rat cortical neurons (purchased from Lonza, Walkersville, MD, USA) were placed on poly-lysine coated glass cover slips in 35 mm dishes, and cultured in primary neuron basal medium with added 2% neural grown factor and 1% glutamine (from Lonza). The medium was half changed every 3 days, and the cells were used at 6–12 days. The neurons had dendrites and axons and the diameters of the somata were about 15–20 µm. Cells were cultured on cover slips and were immersed in 35 mm Petri dishes filled with room temperature extracellular saline. Patch pipettes were pulled from borosilicate glass capillaries with filament (BF150-86-10, Sutter Instrument, Novato CA, USA) on a P-1000 Flaming/Brown micropipette puller (Sutter Instrument). Extracellular solution was the same regardless of the target ion channel and contained: 140 mM NaCl, 5 mM KCl, 2 mM MgCl_2_, 2 mM CaCl_2_, 10 mM glucose, and 10 mM HEPES (pH = 7.4). Intracellular patch solution for potassium channel recordings consisted of: 140 mM KF, 10 mM NaCl, 2 mM MgCl_2_, and 10 mM HEPES (pH = 7.2). Intracellular patch solution for sodium channel recordings consisted of: 140 mM CsCH_3_SO_3_ 10 mM NaCl, 2 mM MgCl_2_, 10 mM HEPES (pH = 7.2), where CsCH_3_SO_3_ was used to eliminate potassium currents during sodium channel analysis. The patch electrodes had resistances of 5–7 MOhms.

Patch clamp recordings were performed with a 40x/0.80W water immersion objective (working distance 3.5 mm) using a forced-air-cooled Photometrics Evolve 512/CCD97 camera system. Following gigaseal formation, the membrane was ruptured through a brief, manual suction of the electrode, or by a 2 msec long zap pulse. Currents were amplified with the use of a patch-clamp amplifier (Axopatch 200B, Molecular Devices LLC, Sunnyvale, CA, USA) and processed for analysis via A/D-converter (Digidata 1440A, Molecular Devices LLC). Recordings underwent series resistance compensation, low-pass filtering at 1 kHz, and were sampled at 10 kHz or 100 kHz for potassium and sodium currents, respectively. For recording and analyzing data, pClamp 10.0 software (Molecular Devices LLC) was used.

## Results

### Lethality of DEET

Insect bioassays showed DEET to be moderately toxic via surface contact and topical application (Table 1). No significant differences were observed between mosquito species, but some statistical difference was observed between the G3 and AKRON strains, indicating little species variability and cross-resistance to DEET in the AKRON strain of *An. gambiae* that carries multiple resistance mechanisms [14], [15]. For comparison, the LD_50_ of the carbamate anticholinesterase propoxur to *Aedes aegypti* was 1.4 (95% CI: 0.9–2.1) ng/mg body weight, approximately 1000-fold less than the LD_50_ of DEET. Co-treatment with the cytochrome P450 monooxygenase inhibitor, piperonyl butoxide (PBO, 200 ng) [23] had no statistically significant influence on the topical toxicity of DEET, and synergism ratios were near unity (Table 1). DEET was also toxic to mosquitoes through surface contact (*i.e*., tarsal) exposure in the WHO paper assay (Table 1). DEET was non-toxic to *Drosophila melanogaster* in a sugar-feeding assay at 1 mg/ml.

**Table 1:** Toxicity values of DEET to three mosquito strains and the housefly.

	aLC_50_, mg/ml	bLD_50_, ng/mg	cLD_50_, ng/ml (+PBO)	
Species	(95% CI)	(95% CI)	(95% CI)	dSR
*A. gambiae*. (G3)	1.9 (0.9–2.9)a	1175 (988–1361)a	1021 (829–1231)a	1.2
*A. gambiae* (AKRON)	2.7 (1.0–4.5)a	1472 (1136–1806)b	1247 (872–1526)a	1.2
*Ae. aegypti*	2.3 (1.1–3.6)a	1102 (836–1367)a	1082 (841–1310)a	1.0
*M. domestica*	–	8104 (7026–9000)c	6219 (4405–8563)b	1.3

Letters after 95% confidence intervals (CI) represent statistical significance for LD_50_ or LC_50_ values among species within a given toxicity measurement column. Values not labeled by the same letter represent statistical significance (P<0.05) in one-way ANOVA followed by Tukey's multiple comparison test.

a LC_50_ in the WHO filter paper assay.

b LD_50_ by topical application in ng of compound/mg of mosquito weight.

c LD_50_ (same units) by topical application after 4 hr pretreatment with 200 ng PBO.

d Synergism ratio  =  LD_50_ with DEET alone/LD_50_ with DEET + PBO.

### Signs of Intoxication

After treatment with lethal doses of DEET, *Aedes aegypti* mosquitoes were found to display both hyperexcitatory and lethargic tendencies. Five minutes after treatment, the mosquitoes were lethargic, with the majority standing with splayed posture or laying ventral side upward. The mosquitoes were unable to rest on the vertical sides of the chamber and collected on the bottom. Agitation induced hyperexcitation, with increased wing beat frequency, spinning on their dorsal side, and erratic movements. Directed flight behavior did not occur. After approximately 10 seconds of hyperexcitation, the mosquitoes resumed lethargic behavior. Control mosquitoes had normal posture (legs not splayed away from the midline), would rest on the sides of the holding chamber versus the bottom, and upon agitation, the mosquitoes would immediately fly from their resting posture to a different location on the container and come to rest.

Mosquitoes intoxicated with propoxur exhibited intense hyperexcitation in the absence of any physical stimulation. Five minutes post-exposure to 1 ng/insect, the mosquitoes were in a supine position and were rapidly beating their wings. This high wing beat frequency caused the mosquitoes to spin on their dorsal side. If the mosquitoes stopped beating their wings, they rapidly twitched and contracted their legs. The mosquitoes continued this behavior until death. Manual agitation caused no change in the behavior of the mosquitoes.

Houseflies presented similar signs of intoxication by DEET, but the flies were mostly lethargic with only occasional bouts of excitation. Unlike *Aedes aegypti*, the flies did not present a change in behavior after agitation, as they remained standing, albeit with splayed posture, and did not begin convulsing. Although the flies presented excitation via twitching and uncoordinated movements, it was not to the same level of intensity as that observed in mosquitoes at toxicologically equivalent doses.

### Anticholinesterase Actions of DEET

DEET was found to be a poor inhibitor of *Drosophila melanogaster* AChE (*Dm*AChE), human AChE (*h*AChE), and *Musca domestica* AChE (*Md*AChE) with mean IC_50_ values in the low milliomolar range (Table 2). The mosquito enzymes, wild type *Anopheles gambiae* AChE (*Ag*AChE) and *Aedes aegypti* AChE (*Ae*AChE), were completely insensitive to DEET at concentrations up to 10 mM, but were highly sensitive to propoxur, with IC_50_ values in the 100–500 nanomolar range for all enzymes except AKRON *Ag*AChE (Table 2). The mean IC_50_ for propoxur to *Md*AChE was 40,000-fold more potent than the IC_50_ value of DEET to *Md*AChE. Lidocaine was found to have an IC_50_ value for *Md*AChE similar to that of DEET. Toluene was inactive at concentrations up to 10 mM on all enzyme preparations.

**Table 2:** AChE inhibition data expressed as mean (n = 3) IC_50_ values.

	IC_50_, nM (95% CI)	IC_50_, mM (95% CI)		
Enzyme	Propoxur	DEET	Lidocaine	Toluene
*An. gambiae* (G3)	447 (420–480)	>10	>10	>10
*An. gambiae* (AKRON)	>100,000	>10	>10	>10
*Aedes aegypti*	370 (320–410)	>10	>10	>10
*Musca domestica*	130 (110–160)	6 (3–8)a	4.9 (3–7)	>10
*Drosophila melanogaster*	84 (69–110)	10 (6–13)b	9.6 (5–14)	>10
Human	1442 (1255–1629)	12 (7–17)b	–	>10

Inhibition values of *Md*AChE, *Dm*AChE, and hAChE were analyzed by one-way ANOVA followed by Tukey's multiple comparison test. Letters after 95% confidence intervals (CI) represent statistical significance for IC_50_ values among enzymes tested with DEET. IC_50_s for DEET not labeled by the same letter represent statistical significance (P<0.05).

### CNS Recordings from Housefly Larvae

**Figure 2:**
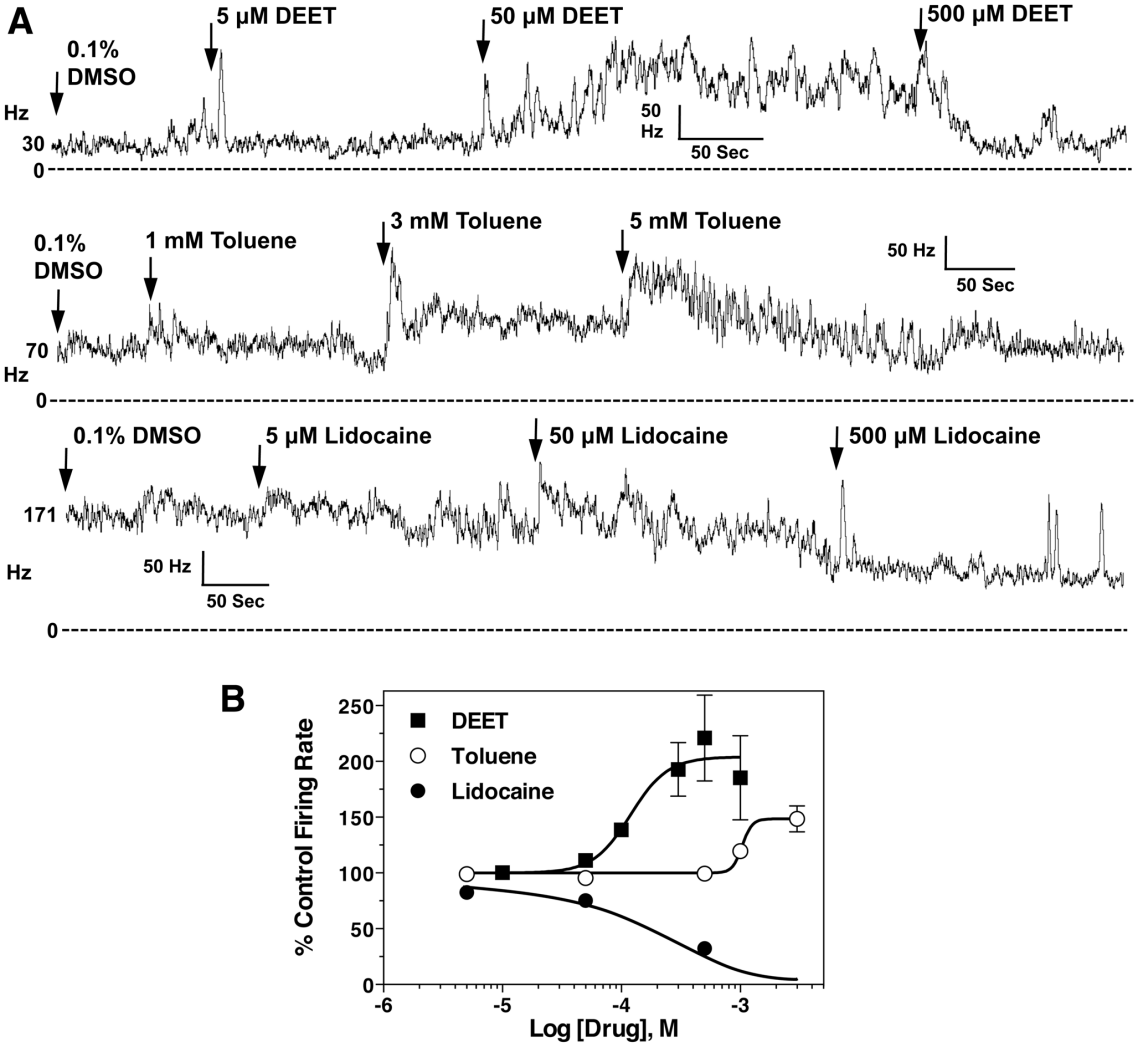
Neurophysiological recordings from the CNS of third instar larvae of *M.* domestica A) Nerve discharges before and after DEET, toluene, and lidocaine treatment across different CNS preparations, as indicated. Initial firing frequencies in spikes/second (Hz) for each experiment are given to the left of each trace. B) Concentration-response curves for DEET, toluene, and lidocaine on CNS nerve discharge of *M. domestica* larvae from replicated recordings (n = 4–5 preparations per curve, with each concentration replicated 4 times), as shown in A. Data points represent mean percentage increase of baseline firing rate, and error bars represent SEM of drug concentrations replicated at least 3 times. When error bars are absent, it is because they are smaller than the size of the symbol.

House fly larval CNS recordings were performed in an effort to further characterize DEET neurotoxicity (Fig. 2). Figure 2A shows representative plots of neuronal discharge rate over time in response to solvent (0.1% DMSO) and treatments with DEET, toluene, and lidocaine. Concentration-response plots of replicated recordings are shown in Figure 2B. DEET was neuroexcitatory with a threshold concentration of about 50 µM, and had EC_50_ values of 0.12 (0.05–0.29) mM and 0.21 (0.08–0.56) mM on transected and intact CNS, respectively. Despite having documented sodium channel-blocking properties [12], toluene was also found to have excitatory effects on nerve firing, but at concentrations about ten-fold higher than that of DEET (Fig. 2A). The EC_50_ of toluene was 1.0 (0.05–24) mM on the transected CNS (Fig. 2B), where the low potency and efficacy of this compound made quantification difficult. Lidocaine, a prototypical local anesthetic, was found to have an inhibitory effect on the CNS (Fig. 2A) with an EC_50_ of 0.34 (0.22–0.54) mM (Fig. 2B).

**Figure 3:**
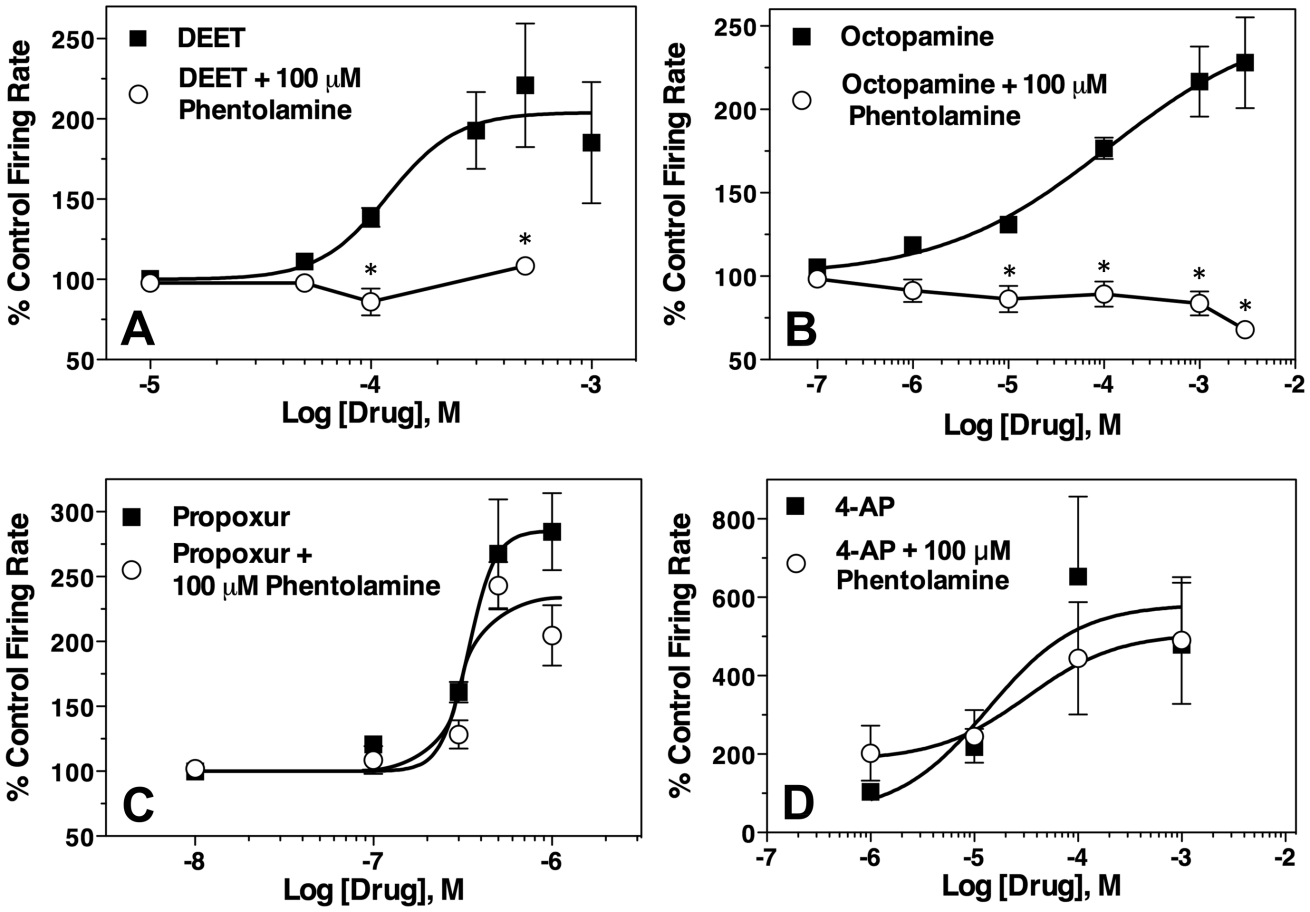
Effect of phentolamine on the activity of DEET (A), octopamine (B), propoxur (C) and 4-AP (D) on discharge rates of housefly larvae CNS preparations (n = 3–5 preparations per curve, with each concentration replicated 3–5 times). Data points represent mean percentage increase of baseline firing rate, and error bars represent SEM of drug concentrations replicated at least 3 times. When error bars are absent, it is because they are smaller than the size of the symbol. Data points at each concentration for drug alone were compared to drug + phentolamine, and statistical significance (t-test, P<0.05) is indicated by an asterisk.

DEET-mediated neuroexcitation and its sensitivity to the octopaminergic antagonist phentolamine was compared to that of octopamine, propoxur, and the potassium channel blocker 4-AP), to assist in the identification of its mode of action. All the compounds increased the maximal firing frequency 2- to 3-fold above control levels (Fig. 3A–C), except for 4-AP, which caused a 6-fold increase (Fig. 3D). Octopamine (Fig. 3B) was found to have an excitatory EC_50_ of 0.11 (0.003–3.8) mM, nearly identical to the EC_50_ found for DEET. Propoxur (Fig. 3C) was used as a positive control for AChE inhibitors, and was found to have an EC_50_ of 344 (251–472) nM for increasing spike frequency in the CNS, 349-fold more potent than that of DEET. The EC_50_ for 4-AP was 13 (0.5–341) µM, and firing declined precipitously at concentrations >1 mM, accounting for the wide confidence limits. Experiments were then performed with phentolamine, an established octopaminergic receptor antagonist [10], to determine its ability to block the neuroexcitation of DEET and test whether neuroexcitation occurred via central octopaminergic pathways. DEET-mediated neuroexcitation (up to 500 µM) was completely blocked by 100 µM phentolamine (Fig. 3A), which also completely blocked octopamine-mediated neuroexcitation at octopamine concentrations up to 3 mM (Fig. 3B). In contrast, 100 µM phentolamine had no effect on the neuroexcitation by propoxur, until the carbamate reached a concentration of 1 µM, where maximal firing declined by 21% compared to propoxur alone (Fig. 3C). This effect was not statistically significant. Similarly, there was no effect of phentolamine on 4-AP mediated neuroexcitation (Fig. 3D).

### Activation of the Firefly Light Organ

**Figure 4:**
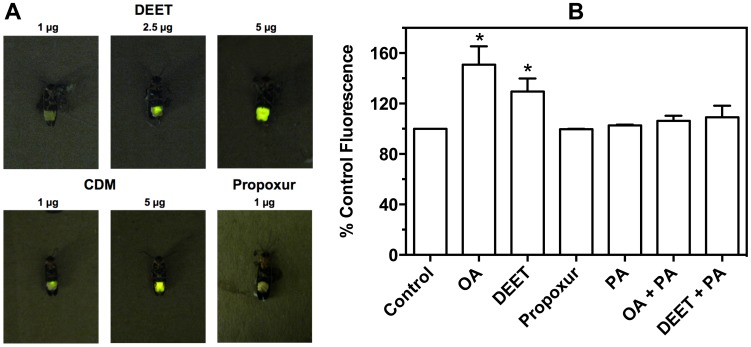
Effects of DEET on octopaminergic systems in firefly and *Sf*21 cells. A) Dose-dependent action of DEET, CDM, and propoxur on the light organ of the firefly, *Photinus pyralis*. See text for explanation. B) Activation of an octopamine receptor in *Sf*21 cells shown by internal calcium fluorescence. Bars represent means of normalized fluorescence with error bars denoting SEM, replicated across individual plates of cells (n = 3). Statistics for each column were determined by a paired t-test against matched control raw fluorescent values, where an asterisk represents statistical significance at P<0.05. For labels, OA =  octopamine and PA =  phentolamine. All compounds were applied at 100 µM.

An octopaminergic action of DEET was next assessed on the firefly light organ (Fig. 4). Control fireflies (1 µL of absolute ethanol) displayed uniformly dark lanterns with intermittent and brief moments of luminescence. The periods of luminescence of control animals were short in duration (*ca*. one second), involved the whole light organ, and flashed every 45-90 seconds. DEET was found to cause dose-dependent activation of the firefly lantern through increased duration and repeated activation of the lantern every 10–20 seconds. Faint illumination in circumscribed areas of the lantern was observed at 1 and 2.5 µg of DEET (Fig. 4A). Bright and complete illumination of the lantern was observed with DEET concentrations of 5 µg and it typically remained active for periods of 10–60 seconds in the majority of individuals. In two individuals, the lantern stayed illuminated for 5 and 30 min periods. At low doses (1 µg, Fig. 4A), the octopaminergic pesticide CDM showed incomplete activation of the light organ, similar to DEET. At higher doses (5 µg, Fig. 4A), the lanterns of CDM treated insects displayed a bright, constant glow of the entire lantern for approximately four hours, after which it began to fade in brightness. In fireflies treated with 1 µg propoxur, typical anticholinesterase signs of intoxication (e.g., tremors and hyperexcitation) were observed, but there were no changes in lantern illumination and propoxur-treated insects flashed their lanterns the same as controls (Fig. 4A).

### Action of DEET at Octopamine Receptor in Sf21 Cells

In order to determine whether DEET had any direct effects on octopamine receptors, it was tested in a cell-based calcium fluorescence assay (Fig. 4B). In these experiments, 100 µM DEET induced a statistically significant (P<0.05) increase of ca. 30% in calcium fluorescence in *Sf*21 cells. Similarly, octopamine caused ca. 50% increase in calcium fluorescence over control cells. For comparison, triton X-100 (0.1%) treatment, expected to flood the cell with calcium via membrane disruption, increased fluorescence 211+12% of control. DEET- and octopamine-mediated activation of calcium fluorescence was abolished by pre-exposure of the cells to phentolamine, yielding fluorescent values not statistically different from controls. These data confirm DEET can activate the octopamine receptor, although it was a weaker agonist than octopamine. As expected, propoxur was shown to have no effect on calcium fluorescence (Fig. 4B).

### Neuromuscular Junction Recordings from Housefly

**Figure 5:**
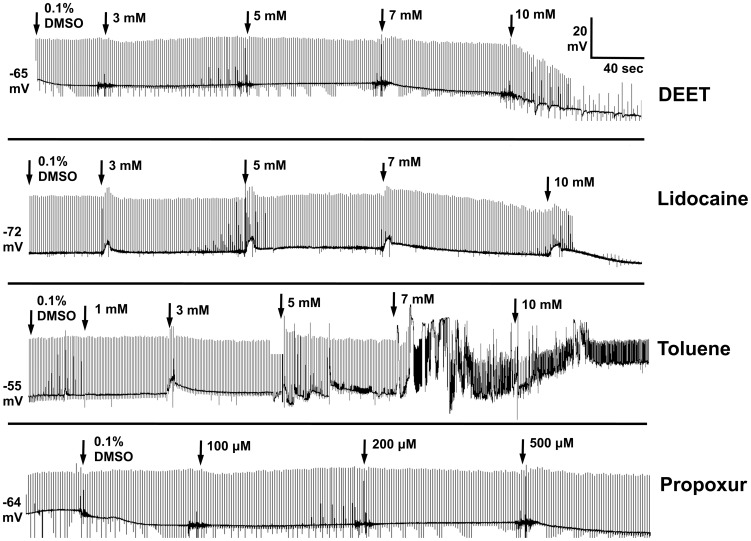
Recordings of the electrically-evoked EPSPs at the neuromuscular junction in *M. domestica* larvae after exposure to DEET (n = 11), lidocaine (n = 8), toluene (n = 8), and propoxur (n = 5). In the DEET and lidocaine traces, the remaining transients after block of the EPSP are stimulus artifacts, which are also reflected by any negative excursions from baseline in all traces (artifact amplitudes were truncated from the recordings for clarity of display).

DEET showed a complete and rapid block of the evoked EPSP in the body wall musculature of third instar *Musca domestica* larvae (Fig. 5). The block was found to be an essentially all or none response, with half of the preparations blocked at ca. 7 mM. Complete block was obtained approximately 120 seconds after 10 mM DEET was added to the bath (Fig. 5). Little or no change was observed in the muscle membrane resting potential after the addition of DEET at any concentration. Lidocaine was also an antagonist at the neuromuscular junction and the blocking pattern was an all or none response very similar to DEET (Fig. 5). Toluene was again found to be excitatory at 5 mM (Fig. 5), and the excitatory effects (spontaneous EPSPs and muscle depolarization) were reversed by washing the preparation with drug-free buffer (data not shown). Propoxur behaved as a negative control, and had no effect on the evoked EPSP of *Musca domestica* larvae (Fig. 5).

### Patch Clamp of Rat Cortical Neurons

**Figure 6:**
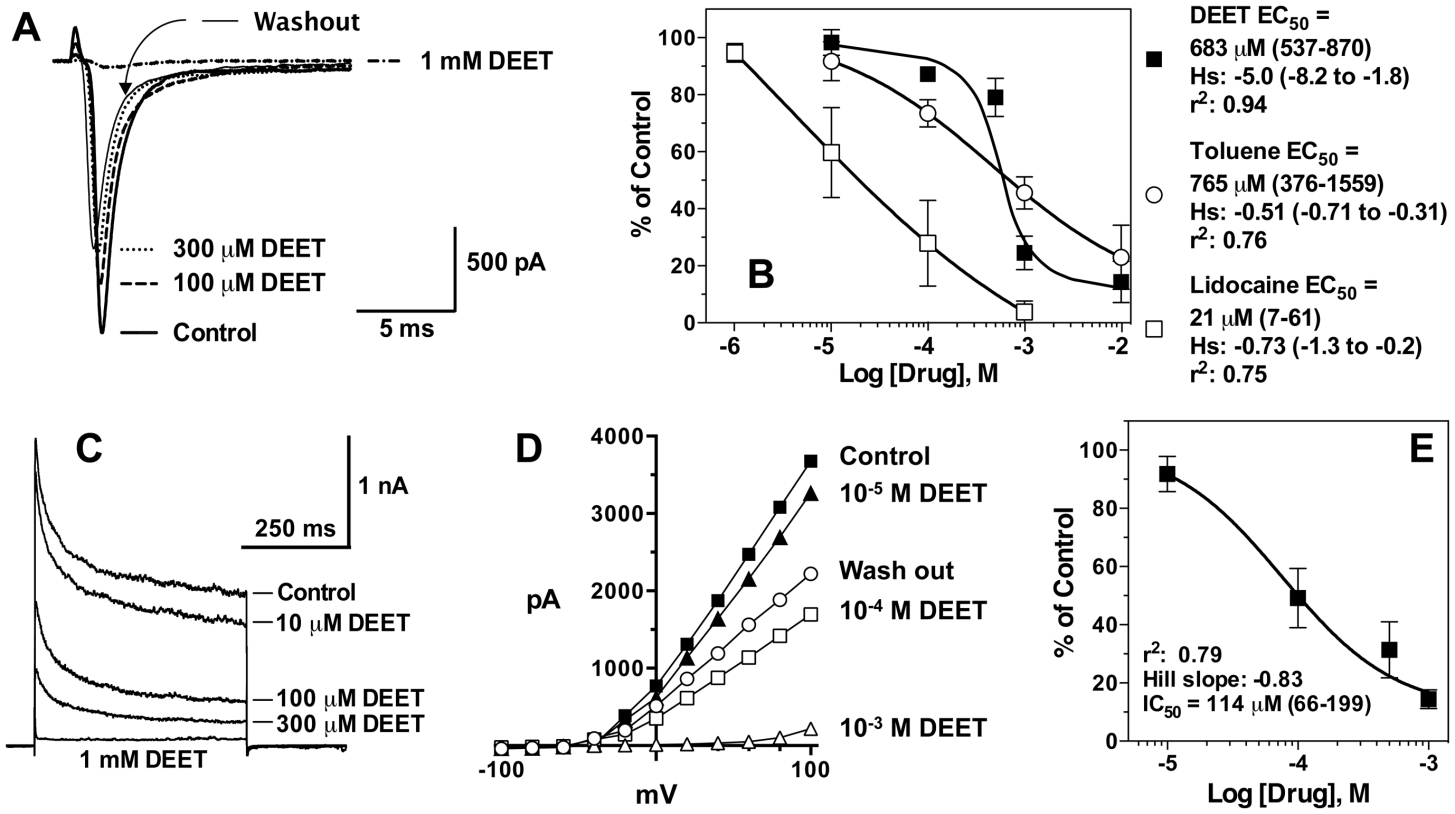
Block of neonatal rat cortical sodium and potassium channels by DEET and related compounds. A) Inward sodium currents activated by a 15 msec pulse to 0 mV from a holding potential of −80 mV. Control is the peak inward sodium current before treatment. Increasing DEET concentrations blocked the current, each trace taken after 1 min incubation in drug, and there was little change thereafter. Each trace is matched in form to treatment: thick line, Control; thin line, Washout; dashed line, 100 µM DEET; dotted line, 300 µM DEET; and dotted/dashed line, 1 mM DEET. B) Concentration-response curves generated from peak sodium currents as shown in A, replicated across different cells treated with DEET (n = 3), toluene (n = 6), or lidocaine (n = 4). Symbols are mean percentage of control current amplitude, with each concentration of blocker replicated 3–6 times. Error bars represent SEM of currents. C) Typical outward currents activated by a 500 msec pulse to +60 mV from a holding potential of −80 mV, and displayed concentration-dependent block by DEET. D) Typical current-voltage relationships and DEET inhibition of potassium currents in rat cortical neurons. Currents were evoked by stepping the membrane voltage between −100 and +100 mV in 20 mV increments from a holding potential of −80 mV. Amplitude of the sustained current was calculated at 200 msec. E) Concentration-response curves for DEET-mediated inhibition of rat neuronal potassium channels from recordings as shown in C, using responses at +60 mV. Symbols are mean percentage of control current amplitude, and error bars represent SEM of currents replicated across different cells (n≥3).

Patch clamp recordings of rat cortical neurons found that DEET is a blocker of both sodium and potassium channels in neonatal rat cortical neurons. DEET completely blocked inward sodium currents at 1 mM, and the effect could be washed out to restore approximately 50% of the sodium current amplitude (Fig. 6A). DEET was found to have an IC_50_ against sodium channels of about 0.7 mM. The DEET-mediated inhibition had similar potency as toluene, but was ca. 30-fold less active than lidocaine. Interestingly, lidocaine and toluene had similar Hill slopes values, while the value for DEET was 7- to 10-fold greater (Fig. 6B). DEET-mediated potassium channel block was also concentration-dependent (Fig. 6C). Current-voltage plots consistent with potassium Kv2 delayed rectifier channels were observed, and DEET blockage was capable of being washed out to restore approximately 50% of control current amplitude (Fig. 5D). Potassium channels were more sensitive to DEET compared to sodium channels, as they were blocked with an EC_50_ that was 6-fold lower (Fig. 6E).

## Discussion

### Whole Animal Toxicity

DEET was found to be toxic at low microgram doses to the mosquitoes *Aedes aegypti* and *Anopheles gambiae*, and to the housefly, *Musca domestica*, which is similar to the mosquito toxicity observed by Corbel et al. [2]. Toxicity to the G3 (susceptible) and AKRON (resistant) strains of *Anopheles gambiae* were nearly identical, indicating that the AChE target site mutation does not protect against the toxicity of DEET. Co-administration of PBO also had no significant impact on the toxicity of DEET, demonstrating that DEET toxicity is not sensitive to P450 monooxygenases. Further, there was no inhibition of mosquito AChE and poor inhibition of *Md*AChE, *Dm*AChE, and human AChE by DEET (Table 2), confirming a previous suggestion that DEET is not an anticholinesterase [7]. These data are noteworthy in light of recent literature [2] reporting *in vitro* enzyme kinetic analyses of *Dm*AChE, as well as human AChE and butyrylcholinesterase. In these studies, DEET showed inhibitory effects at 0.1–10 mM concentrations. The fact that DEET is toxic to the mosquito without measurable inhibition of AChE, *in vitro*, suggests that DEET imposes its neurotoxic effect in a manner other than AChE inhibition. The low potency of DEET as an inhibitor of *h*AChE is consistent with the relatively few reports of cholinergic signs of human intoxication following DEET application, and its high LD_50_ value after oral administration to rats (ca. 3000 mg/kg [24]).

### Housefly CNS Effects

The excitation of the housefly larval CNS after exposure to DEET was different from the response patterns of toluene and lidocaine (Fig. 2A), and besides propoxur, showed no correlation with inhibition of AChE, *in vitro*. Lidocaine was a blocker in all preparations studied, with EC_50_ values similar to those reported in the literature [25], likely indicating a different action on nerve preparations when compared to DEET, regardless of the similar *Md*AChE inhibition potencies for these two compounds (Table 2). The local anesthetic action of lidocaine on sodium channels apparently negates its anticholinesterase action when measured as descending nerve discharges from the CNS. Toluene was uniformly excitatory in all preparations tested, and had no anesthetic action on the insect nervous system. These findings suggest that the local anesthetic binding site of the insect sodium channel or its interaction with toluene is different from that of the rat Na_v_ 1.4 channel, as previous studies found toluene blocked these channels at a concentration of 3 mM, *in vitro* [12], well below the concentrations used in our study.

### Octopaminergic Pathways and Receptors as Targets for DEET

Because DEET had neuroexcitatory effects on the CNS and a chemical structure somewhat similar to CDM, experiments compared the effects of DEET to compounds acting on octopaminergic systems. CDM and octopamine are neuroexcitatory through activation of octopamine receptors, leading to an increased cyclic AMP concentration that enhances neuronal excitability [10]. Phentolamine, a known octopaminergic antagonist [10], completely blocked the CNS neuroexcitation of DEET and octopamine, but was ineffective against neuroexcitation caused by propoxur or 4-AP. These results provide the first direct evidence that DEET targets octopaminergic nerve pathways as the mechanism of neurotoxicity.

Although there is no documented presence of octopamine receptors in *Sf*21 cells, octopamine receptors coupled to adenylate cyclase are present in *Sf*9 cells and respond positively to the demethylated metabolite of CDM [22]. Our data (Fig. 2B) demonstrate the presence of octopamine receptors on *Sf*21 cells linked to internal calcium fluorescence. Both DEET- and octopamine-mediated fluorescence were antagonized by the receptor antagonist phentolamine, similar to the larval CNS recordings. The action of DEET on calcium fluorescence via an octopamine receptor provides a third piece of evidence suggesting an action on octopaminergic systems. These data, along with the poor anticholinesterase action of DEET, provide strong evidence that DEET is primarily targeting octopamine receptor activation and not acetylcholinesterase to induce insect toxicity.

Activation of the firefly (*Photinus pyralis)* light organ further support that DEET is targeting the octopaminergic system, since it is well documented that octopamine is responsible for induction of luminescence within the light organ [26], [27] via cyclic AMP production [28], [29]. CDM is a potent effector of the firefly light organ [30], but complete activation of the lantern is not observed until four hours post-exposure due to the need for CDM to be demethylated, *in vivo*, to its agonist form [30]. DEET activated the firefly lantern in a dose-dependent manner (Fig. 4A), but its luminescent action was slightly different from that of CDM. DEET-induced illumination was less potent and effective than CDM, perhaps reflecting low receptor affinity, desensitization, or another mechanism(s) of action on the lantern. In contrast, the anticholinesterase propoxur at the intoxicating dose used had no effect on the firefly lantern.

### Neuromuscular Actions of DEET

The insect neuromuscular junction was used to assess the action of DEET on the peripheral nervous system and the inactivity of propoxur serves as a negative control for effects of suspected anticholinesterases, as insects lack peripheral cholinergic synapses [31], [32]. DEET blocked the evoked EPSP, an effect consistent with an anesthetic-like action, but was 60–fold less potent than its excitatory action on the CNS. Lidocaine displayed a blocking pattern similar to DEET, indicating a local anesthetic effect and a shared blocking action on the neuromuscular junction. Effects of DEET on the neuromuscular junction were inconsistent with potassium channel block, as there was no evidence of multiple EPSP discharges or any depolarization of the muscle membrane potential, even though four potassium channel subtypes, including calcium-activated, inactivating, and delayed rectifier, are known to exist in these muscles [33]. Perhaps the high concentration of DEET and lidocaine required for block of neurotransmission indicates effects on sodium and potassium channels acting in opposition to each other, or possibly blockage of calcium channels [11], but more experiments would be required to address these possibilities.

### Actions of DEET on Mammalian Na^+^ and K^+^ Channels

Patch clamp studies were performed with rat cortical neurons to determine the mode of action of DEET on mammalian ion channels. DEET was capable of blocking sodium and potassium (Kv2, delayed rectifier type) currents at low- to mid-micromolar concentrations, similar to lidocaine and other local anesthetics. However, for local anesthetics, sodium channel block is more potent than potassium channel block [11], whereas DEET showed the opposite (Fig. 6). In any event, these actions may play a role in the numbing sensation observed when DEET contacts human skin [5]. Local anesthetics have a well characterized numbing effect caused by a blocking action on nerve membrane sodium channels, and this effect is more potent and functionally significant than its ability to block potassium channels, which simply results in a broadening of the action potential [11]. Much further investigation of its ion channel blocking mechanisms is warranted, and should include studies of mammalian sensory nerve conduction in the presence of DEET. DEET blockage of sodium currents had 7- and 10-fold greater Hill slope than lidocaine or toluene, respectively (Fig. 5), but unusually steep Hill slopes were not observed for inhibition of AChE activity or potassium currents. Compounds having steep inhibitory Hill slopes at high concentrations *in vitro*, can occur if there are several inhibitor binding sites on a protein, when [enzyme] >> than inhibitor K_d_, or phase transitions/precipitation/colloid formation occurs for some compounds in high throughput screening situations [34]. Additional research is required to address these different possible mechanisms.

With regard to insect toxicity, it is unlikely that sodium or potassium channel blockage is the primary mechanism of DEET toxicity, since excitatory effects on the CNS are much more potent, and the effects of 4-AP, a classical potassium channel blocker, are not inhibited by phentolamine. This line of reasoning supports the idea that splayed posture and hyperexcitation after toxic doses of DEET arise because of a combination of central octopaminergic hyperexcitation complexed with incomplete suppression of peripheral neurotransmission.

## Conclusions

DEET is of relatively low acute toxicity to insects and mammals. It is unlikely that DEET exerts its toxicity through anticholinesterase action because of its low potency for enzyme inhibition and the complete block of its neuroexcitation in housefly CNS by phentolamine, which does not occur with propoxur or 4-AP. DEET toxicity to houseflies, and likely mosquitoes, is mediated by central octopaminergic pathways via activation of octopamine receptors. The poisoning signs caused by DEET reflect an excitatory octopaminergic effect in combination with peripheral neurosuppressive actions. The low potency of DEET for inhibiting human acetylcholinesterase makes it unlikely to cause toxicity by this mechanism. Numbness of mammalian mucous membranes is explained through anesthetic-like effects of DEET on nerve conduction.
